# Supplementary material for: Factors associated with the enrollment of commercial medical insurance in China: Results from China General Social Survey
Source: PLoS One. 2024 May 23;19(5):e0303997. doi: 10.1371/journal.pone.0303997 (PMC11115273; doi:10.1371/journal.pone.0303997)
Supplement: S1 File — (DOCX) [file pone.0303997.s001.docx]

**The selection of the relevant data, the detailed measurement of the independent variables and how they were coded**

Based on the principal-agent framework, relevant data was selected from eight sections of the questionnaire, including (a) sociodemographic characteristics (e.g. age, gender, education), (c) health status (e.g. self-assessed physical health), (d) migration status (e.g. household registration status), (e) lifestyle behaviors, (g) class identity, (j) participation of labor market, (k) social security status, and (l) family characteristics (e.g. household size). The key variable of study interest was the enrollment rate of the CMI. A total of 18 independent variables were included in the current study.

**1. Explicit characteristics:**

(1) sociodemographic characteristics

After recoding, the sociodemographic variables of this study include: Sex(Male = "1",Female = "2"),Age(continuous variables, calculated by year of survey minus year of birth),Marital status(Married=”1”,Widowed=”2”,Divorced=”3”, Unmarried=”4”),Education(Primary school and below=”1”, Junior high school=”2”, High school or secondary school =”3”, Junior college and above=”4”), Number of family members (continuous variables, adjust for the number of all household members reported in the questionnaire), Current work experience and status (Engage in non-agricultural work=”1”,Farming=”2”,Not in employment=”3”),Household registration status(agricultural household=”1”, Non-agricultural household =”2”),Subjective social status (Lower=”1”,Middle=”2”,Upper=”3”).

(2) family economic status

The variables in this part mainly include: Number of family houses (continuous variables, how many properties the family has in total), Have a car (No=”1”, Yes=”2”), Investment activities (No=”1”, Yes=”2”), Level of family economy (Average=”1”, Below average=”2”, Above average=”3”).

**2. Latent characteristics: social security status**

Participate in basic medical insurance (Yes=”1”, No=”2”), Participate in basic pension insurance (Yes=”1”, No=”2”) are selected in this section.

**3. The global incentive: health status**

The health status variables mainly include BMI, self-assessment of physical health, has been hospitalized due to illness or injury in the past year, frequency of physical exercise. We recoded them as follows. BMI (adopt Chinese standard, Normal range=”1”, Above normal range=”2”, Below normal range=”3”), Self-assessment of physical health (Healthy=”1”, Average=”2”, Unhealthy=”3”), Has been hospitalized due to illness or injury in the past year (No=”1”, Yes=”2”), Frequency of physical exercise (Never=”1”, Sometimes=”2”, Every day=”3”).
