# Supplementary material for: Factors associated with the enrollment of commercial medical insurance in China: Results from China General Social Survey
Source: PLoS One. 2024 May 23;19(5):e0303997. doi: 10.1371/journal.pone.0303997 (PMC11115273; doi:10.1371/journal.pone.0303997)
Supplement: Attachment — Submitted filename: PONE-D-23-25788.pdf [file pone.0303997.s003.pdf]

China has a vast territory and a large population, and promoting commercial medical insurance (CMI) can indeed play a certain role in solving disease burden, health inequalities and other health care challenges.

The author/ authors used Mann-Whitney U test and Chi-square test, and Binary logistic regression analysis to analyze the data from the 2018 Chinese General Social Survey (CGSS), with the purpose of exploring factors related to CMI enrollment, including sociodemographic characteristics, health status, and family economic status. and social security conditions to provide reference for the design and development of China's CMI.

However, the paper suffers from several flaws, some of which appear to be significant, greatly reducing the value of this study. Therefore, I offer the following advice to author/authors.

1. Discussing the factors related to CMI enrollment can provide important policy directions when promoting CMI. There are many relevant published literatures, but I have not seen the author/authors doing a review in this area, which is a pity. It is recommended that author/authors read journal articles published in Journal of Risk and Insurance or Geneva Risk and Insurance Review. Because various studies are progressive in their contributions, the author/authors can consider whether it is necessary to introduce category theory, deductive generation, causal classification, etc., but the author/authors still has to make proof and efforts in this regard.

2. This study used data from the 2018 CGSS. Can the author/authors explain why there is only one year of data? Because the CGSS data has been launched since 2003. If multi-year data were available, the author/authors could conduct a richer analysis and discussion.

3. The author used Mann-Whitney U test and Chi-square test, and Binary logistic regression analysis in the analysis. Mann-Whitney U test and Chi-square test can only analyze whether there are significant differences in some relevant variables between the two groups of CMI-enrollees and CMI non-enrollees. Binary logistic regression analysis analyzes the impact of relevant variables on the decision-making of CMI-enrollees and CMI non-enrollees of the tested samples. I think the author's choice of research methods was relatively simple due to insufficient reading of the research literature, which made this study look like a statistical

exercise rather than a research paper. Why did the authors not consider the propensity score matching (PSM)? This method may be more suitable for this study. Likewise, the author/authors can understand the operation and application of this method by reading the research literature.

4. Author/authors must clearly state how the explanatory variables in the analytical model are chosen because this affects the interpretation of the analytical results. Because the data chosen by the author is from China, there may be many obvious differences with data from other countries, such as the start time of the development of commercial insurance, differences in policy regulations, etc. These factors that have not been taken into account will cause the CMI enrollment in China to be different from that in other countries. The author's explanation in this part is insufficient.
