# Supplementary material for: Factors associated with the enrollment of commercial medical insurance in China: Results from China General Social Survey
Source: PLoS One. 2024 May 23;19(5):e0303997. doi: 10.1371/journal.pone.0303997 (PMC11115273; doi:10.1371/journal.pone.0303997)
Supplement: Attachment — Submitted filename: PONE-D-23-25788-R2.pdf [file pone.0303997.s005.pdf]

Thanks to the editor-in-chief and the author for giving me the opportunity to read this paper again. I can clearly feel the author's efforts and the improvement in the quality of the paper. However, I still have some concerns about the current version of the paper and would like to provide the author with suggestions for revision.

1. Regarding the first suggestion I made after the previous reading, I hope the author will introduce necessary category theory, deductive generation, causal classification, etc., because even though this article is an exploratory study, I think many analysis variables are set and proposed. It still requires theoretical basis or inference, and the author's improvement in this part is still insufficient.
2. I still hope that the author will increase the number of data years analyzed instead of only using the data of 2018 for a cross-sectional analysis. I agree that the environmental background of the 2003 data and the 2018 data is very different, but the author can use the data from 5 or 8 years before 2018 and then use the panel model. I would like to emphasize that the conclusions of many studies may be the result of institutional or environmental changes over the years. Therefore, I hope that the authors will consider using multi-year data again on the premise of data availability.
3. I asked the author to review studies in other countries similar to this paper because I hope the author can explore the possible reasons for the differences between China and other countries on some variables. There must be certain social, economic or institutional variables that cause the enrollment of commercial medical insurance in China to be different from that in other countries. Therefore, I suggest that the author still needs to review other studies and provide explanations. Alternatively, the suggestion I made in the first point is also a direction that the author can take. The purpose of doing so is to improve the academic contribution of this paper.
