# Supplementary material for: Factors associated with the enrollment of commercial medical insurance in China: Results from China General Social Survey
Source: PLoS One. 2024 May 23;19(5):e0303997. doi: 10.1371/journal.pone.0303997 (PMC11115273; doi:10.1371/journal.pone.0303997)
Supplement: Attachment — Submitted filename: PONE-D-23-25788-R3.pdf [file pone.0303997.s007.pdf]

I would like to thank the editor-in-chief and the authors again for giving me the opportunity to review this study. The current version has been greatly improved and I will recommend it to the editor-in-chief for publication.

1. The author has added consideration to factors that have been discussed in other literature that affect people's purchase of health insurance, which makes the model more complete.
2. The author has added data from 2015 and 2021, which adds discussion of differences and similarities in different years during the analysis process, allowing readers to understand the changes in factors affecting Chinese people’s health insurance purchase over time.
3. In the end, the author still did not do any comparison of factors affecting health insurance coverage between China and other countries, which is a pity. However, the content of this current version is more complete and richer than the previous two versions. Perhaps the comparison of factors affecting health insurance enrollment in China and other countries can be used as a direction for future extended research.
