# Supplementary material for: Factors associated with the enrollment of commercial medical insurance in China: Results from China General Social Survey
Source: PLoS One. 2024 May 23;19(5):e0303997. doi: 10.1371/journal.pone.0303997 (PMC11115273; doi:10.1371/journal.pone.0303997)
Supplement: Attachment — Submitted filename: Response to Reviewers.docx [file pone.0303997.s006.docx]

Dear Editor and reviewers,

Thank you very much for your comments, which have helped to improve the rigor and quality of our manuscript. Please see below point-to-point responses and changes in the revised manuscript (highlighted in yellow).

Reviewer #1:

The revised paper was improved a lot. About the multivariate logistical regression issue I mentioned last time, I appreciated that that author provided me with a reference about the difference between multivariate/multivariable regression. What I wanted to say last time is that multivariable regression should be taken. In the author’s response, they claimed that “multivariable regression” was adopted. However, in the methods part, “multivariable analysis” was used to describe the groups comparisons but not logistic regression. Authors may misunderstand the term “multivariable analysis”, which does not mean that multiple variables are analyzed independently but are analyzed simultaneously to get combined results for each variable. For instance, when we analyze marital status and enrollment in table 5, we put age and/or other variables in. That is multivariable regression, which can be used to adjust the relationship between one independent variable and enrollment by introducing other variables in analysis. A multivariable regression analysis is recommended for consideration again.

**Response:**

Thanks for the clarification. We used logistic regression models to perform the multivariable analysis. The independent variables included in the logistic regression model are those that meet certain criteria in the bivariate analysis (e.g. Chi-square test and Mann-Whitney U test). The independent variables in the logistic regression were analyzed simultaneously instead of independently. The results are presented in Table 5. We also revised the method section regarding the logistic regression to further clarify it. The revised sentences are “To explore the association of potential factors associated with enrollment of CMI, a multivariable logistic regression model was used. The dependent variable was whether to enroll in CMI (Yes=1, No=0). In this revised submission, the independent variables were explicit characteristics (sociodemographic characteristics and family economic status), latent characteristics (social security status), and the global incentive compatibility index (health status). The independent variables with a p-value less than 0.2 in the Mann-Whitney U test and Chi-square test were included in the subsequent binary logistic regression analysis. The results were presented as adjusted ORs with 95% confidence interval (CI). The significant level was set at 5%”

(Please see pages 9-10 of the revised manuscript, lines 155-161.)

Reviewer #3:

Thanks to the editor-in-chief and the author for giving me the opportunity to read this paper again. I can clearly feel the author's efforts and the improvement in the quality of the paper. However, I still have some concerns about the current version of the paper and would like to provide the author with suggestions for revision.

1. Regarding the first suggestion I made after the previous reading, I hope the author will introduce necessary category theory, deductive generation, causal classification, etc., because even though this article is an exploratory study, I think many analysis variables are set and proposed. It still requires theoretical basis or inference, and the author's improvement in this part is still insufficient.

**Response：**

We appreciate the reviewer’s comments. We introduce the principal-agent model to understand the factors associated with the enrollment of the CMI in China in this revised version. Based on Nganje’s framework on determinants of health insurance according to the idea of the principal-agent model (citation: Nganje W, Addey KA. Health Uninsurance in rural America: a partial equilibrium analysis. Health economics review. 2019;9(1):19. Epub 2019/06/21. doi: 10.1186/s13561-019-0234-x. PubMed PMID: 31218435; PubMed Central PMCID: PMCPMC6734485.), we reviewed the literature on factors associated with health insurance and found that the main potential determinants of health insurance enrollment were primarily categorized into (1) Explicit characteristics, including sociodemographic characteristics(e.g., age, gender, employment, and education) and family economic status(e.g., family size, income, and property); (2) Latent characteristics, such as the existence of competing risk protection mechanisms, can reflect the psychological characteristics of risk-averse (e.g., basic medical insurance); and (3) The global incentive compatibility index, including health-related factors (e.g., lifestyle, and past health experience).

2. I still hope that the author will increase the number of data years analyzed instead of only using the data of 2018 for a cross-sectional analysis. I agree that the environmental background of the 2003 data and the 2018 data is very different, but the author can use the data from 5 or 8 years before 2018 and then use the panel model. I would like to emphasize that the conclusions of many studies may be the result of institutional or environmental changes over the years. Therefore, I hope that the authors will consider using multi-year data again on the premise of data availability.

**Response：**

Thanks for your suggestions. We searched the public data from 5 or 8 years around 2018 on the official website of China General Social Survey (CGSS) and found that data for 2013, 2015, 2017, 2018, and 2021 were available. Considering the availability and consistency of data available for certain variables, and to reflect the patterns of factors associated with enrolment of CMI in China, we chose data for 2015, 2018 and 2021. After removing the missing data, we included 10,305, 11,666 and 7,252 individuals in 2015, 2018, and 2021. We explain this in the “Data sources” section of this article, and the specific description is “We used CGSS in 2015, 2018, and 2021 that contained respondents from 29 provinces/cities/autonomous regions in China. After removing missing data, the datasets in 2015, 2018, and 2021 contain 10,305, 11,666, and 7,252 samples, respectively.”

(Please see page 8 of the revised manuscript, lines 123-126).

After data analysis, the data analysis results of 2015 and 2021 were added to the tables. We found that of all individuals, 8.7%,11.8% and 14.1% had enrolled in CMI in 2015,2018 and 2021, respectively, and the factors associated with CMI were consistent in these three years. Moreover, we found that individuals without basic pension insurance were less likely to enroll in CMI when analyzing the 2015 data. And we also found that obese individuals and divorced individuals were more likely to enroll in CMI when analyzing data for 2021. These findings were added to the “Results” and “Discussion” sections.

The specific description is as follows.

“We found that of all individuals, the proportion of enrolled individuals shows an increasing trend year by year, with 8.7%,11.8% and 14.1% enrolled in CMI in 2015,2018 and 2021, respectively.”

(Please see page 38 of the revised manuscript, lines 272-274).

“Divorced individuals were 1.48 times more likely to enroll in CMI than married individuals (OR=1.48, 95% CI: 1.03-2.13 in 2021).” “Individuals without basic pension insurance were less likely to enroll in CMI compared to those with basic pension insurance (OR=0.80, 95% CI:0.66-0.97 in 2015).” “In perspective to the global incentive compatibility, obese individuals were 1.20 times more likely to enroll in CMI than those BMI within normal range according to the BMI range (OR=1.20, 95% CI: 1.03-1.39 in 2021).”

(Please see pages 30-31 of the revised manuscript, lines 241-242, lines 259-261, lines 262-264).

“However, we also found that divorced individuals were more likely to enroll in CMI than those who were married, which is different from previous study [11]. This may be because divorced individuals who have been through marriage are more convinced of the importance of medical insurance.”

(Please see page 40 of the revised manuscript, lines 316-319).

Cite-[11] - Balqis-Ali NZ, Anis-Syakira J, Weng HF, Sararaks S. Private Health Insurance in Malaysia: Who Is Left Behind? Asia-Pacific Journal of Public Health. 2021;33(8):861-9.

“We also found that obese individuals were more likely to enroll in CMI than those BMI within normal range. As obese individuals have a much higher risk of disease than normal individuals, it may be that obese individuals feel they need CMI to reduce the financial risk of medical treatment.”

(Please see pages 42-43 of the revised manuscript, lines 374-377).

“However, we found that individuals without basic pension insurance were less likely to enroll in CMI compared to those with basic pension insurance. This may be because individuals without basic pension insurance do not pay attention to the role of insurance, and will not spend more money to enroll in CMI.”

(Please see page 42 of the revised manuscript, lines 363-366).

3. I asked the author to review studies in other countries similar to this paper because I hope the author can explore the possible reasons for the differences between China and other countries on some variables. There must be certain social, economic or institutional variables that cause the enrollment of commercial medical insurance in China to be different from that in other countries. Therefore, I suggest that the author still needs to review other studies and provide explanations. Alternatively, the suggestion I made in the first point is also a direction that the author can take. The purpose of doing so is to improve the academic contribution of this paper.

**Response：**

According to your suggestion, we have introduced the principal-agent model in this revised version to understand the factors associated with the enrollment of the CMI in China and analyzed the multi-year data to improve the academic contribution of this paper.

Indeed, there must be certain social, economic or institutional variables that cause the enrollment of CMI in China to be different from that in other countries. We did not analyze these factors as variables because there was no available data on these aspects in the questionnaire we used. However, we added some comparison with other countries in these aspects in the previous manuscript, such as the start time of the development of commercial insurance and differences in policy regulations. The specific description is “However, compared with the United States and some developed European countries, the coverage of CMI in China is relatively low. The differences in coverage may be influenced by factors such as the start time of the development of CMI and policy regulations. For instance, in the United States where the “market-oriented” health insurance model is dominant, 55.3% of individuals were enrolled in CMI in 2016 [34]. The high coverage may be related to the earlier establishment and development of the CMI system in the United States which began around 1945 as a pillar of its medical security system [35], and the strong legal protection for those residents who do not have employer-provided health insurance and do not qualify for public insurance to purchase CMI [36]. Besides, the governments of England, Germany, and the United States have all introduced tax support policies for CMI, which has a positive impact on increasing the CMI coverage.”

(Please see page 38 of the revised manuscript, lines 275-284.)

Cite-[34] - Papanicolas I, Woskie LR, Jha AK. Health Care Spending in the United States and Other High-Income Countries. Jama. 2018;319(10):1024-39. Epub 2018/03/15. doi: 10.1001/jama.2018.1150. PubMed PMID: 29536101.

[35] Rice T, Rosenau P, Unruh LY, Barnes AJ. United States: Health System Review. Health systems in transition. 2020;22(4):1-441. Epub 2021/02/03. PubMed PMID: 33527901.

[36] McIntyre A, Song Z. The US Affordable Care Act: Reflections and directions at the close of a decade. PLoS medicine. 2019;16(2):e1002752. Epub 2019/02/27. doi: 10.1371/journal.pmed.1002752. PubMed PMID: 30807584; PubMed Central PMCID: PMCPMC6390990 following competing interests: ZS is a member of the Editorial Board of PLOS Medicine.
